# Spontaneous rupturing of splenic artery aneurysm: Another reason for fatal syncope and shock (Case report and literature review)

**DOI:** 10.1515/med-2022-0445

**Published:** 2022-03-23

**Authors:** Feng Yuan, Liudang He, Zhengbin Yao, Yong Long, Shugen Xu

**Affiliations:** Department of Emergency Medicine, The Affiliated Changsha Central Hospital, Hengyang Medical School, University of South China, Hengyang, Hunan, China

**Keywords:** splenic artery aneurysm, syncope, shock, POCUS

## Abstract

Splenic artery aneurysm (SAA) is a rare condition; however, it is one of the most common intra-abdominal aneurysm. In the emergency department (ED), due to an uncommon cause of shock and syncope in SAA, it poses great diagnostic challenge for emergency physicians. Here we reported a case of spontaneous rupturing of SAA. A 47-year-old man presented to the ED for syncope and shock. As he had unstable hemodynamic, we gave him fluid resuscitation and point-of-care ultrasound (POCUS), free intraperitoneal fluid was identified on ultrasound, then hemorrhagic ascites was identified by a diagnostic abdominal paracentesis. The rare but life-threatening diagnosis of spontaneous rupturing of SAA was confirmed by contrast-enhanced Computed Tomography and surgery. Spontaneous SAA rupturing is a rare fatal condition which needs immediate diagnosis and management to achieve a favorable outcome. Though there are no risk factors, emergency physicians should consider SAA in the differential diagnosis of sudden collapse. Also, as an emergency physician, it is very important to be a master of first aid skills such as POCUS and treat patients according to the process.

## 1 Introduction

Splenic artery aneurysm (SAA) is a rare potentially fatal condition, the incidence varies from 0.1 to 10.4% in the general population [1]. It is the third most common intra-abdominal aneurysm followed by aortic and iliac arteries aneurysms [2]. With the potential risk for rupture and life-threatening hemorrhage shock, the mortality rate of ruptured SAA is 10–25% in non-pregnant patient and up to 70% during pregnancy [3]. With the advances made in radiologic studies and increased aging population, the diagnoses of SAA are increasing. It is four times more common in females compared to males from the study by Dave et al. [4], but it seems no different in the study by Hamid et al. [5]. Although the pathogenesis is not fully understood, the risk factors include pregnancy, portal hypertension, splenomegaly, medial fibroplasia, liver cirrhosis, liver transplantation, degenerative atherosclerosis, pancreatic pseudocyst, polyarteritis nodosa, vasculitis, and congenital anomalies affecting the arteries of the foregut [6,7]. In our case of spontaneous SAA rupture, the patient didin't have any previously know risk factor.

## 2 Case presentation

**Figure 1:**
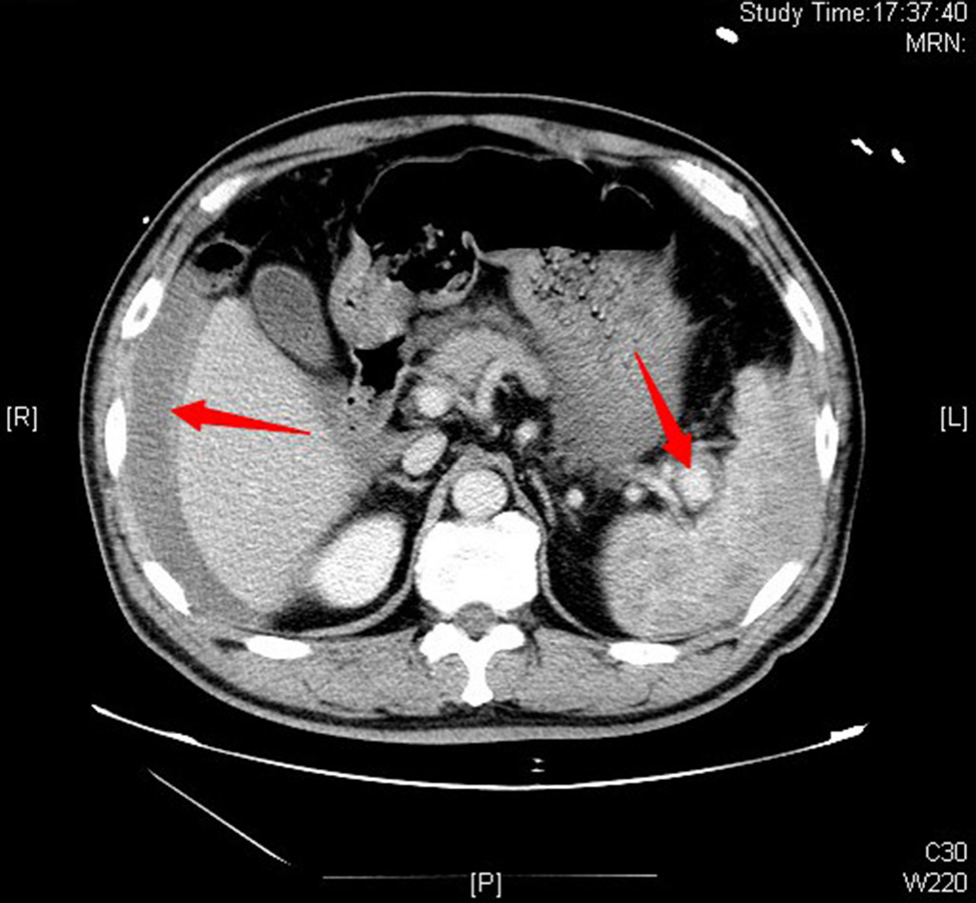
Contrast-enhanced CT of the abdomen showed perihepatic hemorrhage and splenic aneurysm (arrows).

**Figure 2:**
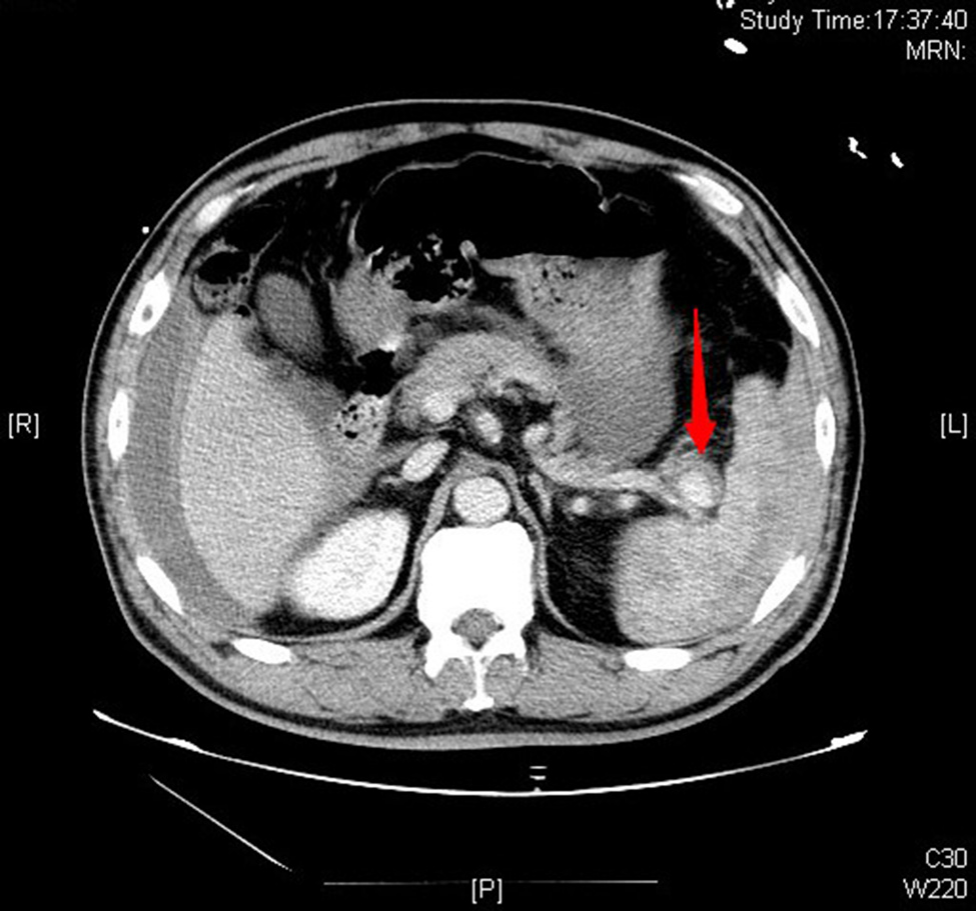
Contrast-enhanced CT of the abdomen showed contrast agent extravasation and ruptured splenic aneurysm (arrow).

**Figure 3:**
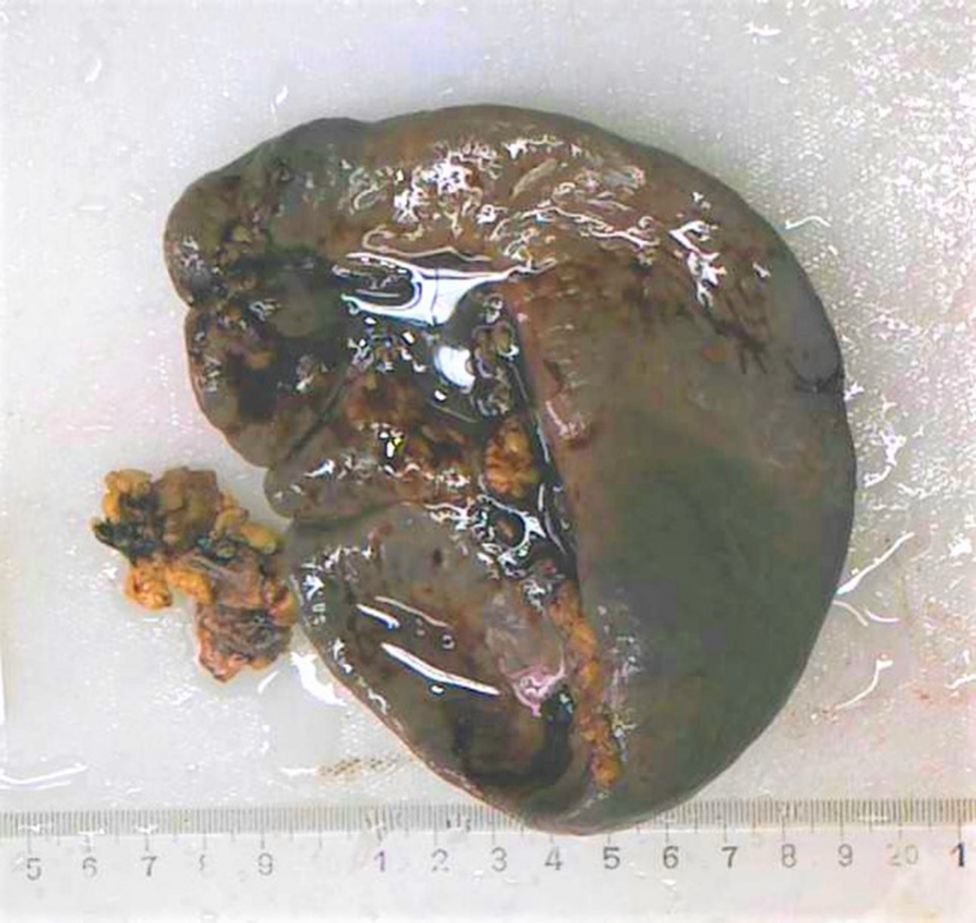
The postoperative pathology report confirmed the diagnosis of a true splenic artery aneurysm.


A 47-year-old male was admitted to our emergency department (ED) by emergency medical services (EMS) due to a sudden syncope and shock 30 min after lunch. There were no associated symptoms nor a history of any disease or trauma. He was conscious during admission, and just felt dizzy and tired with cold sweat. Physical examinations revealed: heart beats 132 per min, blood pressure 82/51 mmHg, respiration rate 20 per min, and transcutaneous oxygen saturation 99%. Neurological examination showed no abnormal findings, also no meaningful findings found in chest and heart examination, and abdomen was soft, epigastric abdominal mild general tenderness, but no rebound tenderness; liver and spleen were impalpable. We thought the syncope was the result of hypovolemic shock, so we gave him fluid resuscitation and point-of-care ultrasound (POCUS), free intraperitoneal fluid was identified on ultrasound, then a diagnostic abdominal paracentesis was performed and hemorrhagic ascites were identified. A contrast-enhanced Computed Tomography (CT) was performed immediately. CT angiogram showed large amount of free intraperitoneal fluid and a SAA with intravenous contrast extravasation consisting of intraperitoneal hemorrhage (Figures 1 and 2). After that, with the consent of the patient and his family, an emergency surgery was performed. A midline laparotomy incision was performed, and a total amount of 3 L blood were evacuated. The splenic artery was ligated proximally, followed by a splenectomy. The patient was transferred to the ICU for unstable hemodynamic after surgery, but was transferred to general ward next day. The patient had recovered and discharged home on the 7th postoperative day. During the 1 year follow-up, the patient held up well without any complications. The postoperative pathology report confirmed the diagnosis of a true SAA (Figure 3).

**Ethics approval and consent to participate:** Since this is a case report, approval from the local ethical committee is not necessary.
**Consent for publication:** The patient has given signed consent for publication of data (including individual details and images).

## 3 Discussion

SAA includes true aneurysms and pseudoaneurysms. The difference between them is that all three layers of the vessel wall where the true aneurysm is located are thinned and dilated, but the pseudoaneurysms result from a tear in the vessel wall which is caused by subsequent formation of a peri-arterial hematoma. Pseudoaneurysms are usually posttraumatic or the result of a local surrounding inflammation [8]. The pseudoaneurysms which lacks one or more layers of the vessel wall, make it weaker and more susceptible to rupture.

The majority of patients with SAA are asymptomatic, which makes it usually diagnostic incidentally [9]. About 20% SAA patients have nonspecific symptoms such as abdominal pain in the epigastrium or left upper quadrant, chest pain, anorexia, nausea, or vomiting. Though the pathogenesis is not fully understood, some risk factors for rupture of the aneurysms are confirmed, which include pregnancy, a diameter greater than 2 cm, portal hypertension, development of symptoms, and liver transplantation et al. [10,11].

SAA rupture results in an active intra-abdominal bleeding and hemorrhagic shock, which could be fatal if not treated on time. It will be much more serious for pregnancy patient, to whom the mortality rate can reach as high as 75% [12]. Even worse, some studies showed that about 95% of SAA ruptures occurred in pregnancy [13]. For diagnosis of ruptured SAA, contrast-enhanced CT, magnetic resonance image, and magnetic resonance angiography were more sensitive and have the advantage of providing three-dimensional images compared to conventional ultrasound [14]. The gold standard for the diagnosis of SAA is digital subtraction angiography (DSA), which can provide the precise location of the aneurysm, assess collateral branches, locate the source of bleeding, and document or exclude other visceral aneurysms simultaneously [4], but the guideline [15] of The Society for Vascular Surgery in 2020 recommends computed tomography angiography as the initial diagnostic tool of choice for SAAs.

The basic managements of ruptured SAA mainly include fluid resuscitation and hemodynamic support, but irrespective of the hemodynamic being stable or not, urgent surgery is needed. Moreover, it is suggested that all symptomatic SAAs should be treated as a matter of urgency [16]. The newest guideline [15] recommends treating nonruptured splenic artery true aneurysms of any size in women of childbearing age because of the risk of rupture. A range of therapeutic options are available to deal with SAA. Open surgical approaches is the first choice for ruptured SAA, but the stenting or coil embolization can also be used for both the ruptured and asymptomatic SAA [8]. Open surgical approaches may include splenectomy with removal of the aneurysm, proximal and distal splenic artery ligation with or without resection of the aneurysm, and trans-aneurysmal arterial ligation [17], which depend on patient’s profile. Partial splenectomy is performed in order to preserve immune function. However some evidence suggests ligation or embolization of the splenic artery also impairs splenic function despite preservation of the organ [18]. The endovascular treatment usually is operated among the patients who are not candidate for surgery or hemodynamic stability. In patients with ruptured SAA diagnosed on preoperative imaging studies, the guideline [15] suggests treatment with open surgical or appropriate endovascular techniques based on the patient’s anatomy and underlying clinical condition.

In our case, the patient was confirmed to be a true splenic aneurysm after surgery, but he had no risk factors, and before the rupture, the patient had no symptoms, even after the splenic aneurysm ruptured, the patient’s symptoms were not typical, this made our timely diagnosis and treatment more difficult. Although the patient finally recovered through surgery, the diagnosis process was full of challenges, and even a little hesitation may have been fatal to the patient. In ED, the most common cause of syncope and shock in non-traumatic patients is craniocerebral disease or heart disease. In the literature reports of many ruptured splenic aneurysms, there are few cases of transient syncope. As the incidence of spontaneous splenic aneurysm rupture is very low, also, emergency patients often fail to provide a detailed medical history, coupled with the crowded ED and lack of resources, all these make it more difficult for emergency physicians to diagnose and provide treatment on time. On the other hand, the ED is often the first department for timely diagnosis for patients with ruptured splenic aneurysm, and treatment greatly affects the prognosis of patients; therefore, it is particularly important to improve emergency physicians’ awareness of ruptured splenic aneurysms and standardize the emergency diagnosis and treatment process.

From this case, we can also see that the applications such as POCUS or Focused Assessment with Sonography for Trauma (FAST) in the ED can provide timely and efficient diagnosis and treatment by doctors and significantly improve the prognosis of patients. POCUS, with the advantages of speed and convenience, does not require to move the patient and it is easy to operate. The process can be completed in a few minutes and has high sensitivity. It can provide effective information for the treatment of emergency patients, especially for trauma or shock patients, when effusion is found in the liver and kidney crypts, spleen and kidney crypts, and pelvis, the damage to the corresponding parts can be found in time, thereby providing guarantee for timely and rapid rescue of critically ill patients. In this case, it was precise because of POCUS’ timely detection of the effusion and initial understanding of the cause of the patient’s shock, enabling treatment to the patient in a timely and effective manner, avoiding adverse consequences.

## 4 Conclusion

SAA rupture is a rare and fatal condition, and not providing timely treatment often imperils the patient’s life. For ruptured SAA, the key to successful treatment lies in early detection. The ED is the first department to take action when a patient arrives at the hospital, so when patient is presenting with abdominal pain, syncope, and signs of hypovolemia, the diagnosis of ruptured SAA should be considered by emergency physicians. Delay in prompt treatment is detrimental to patient’s survival; also, it emphasizes the role of emergency diagnosis and treatment process and easily available diagnostic modalities such as POCUS. The limit of this study is that only one case of emergency treatment of a ruptured SAA is reported, more research needs to be further carried out, which is conducive to the standard emergency treatment of such patients.

## Abbreviations

DSA: digital subtraction angiography
ED: emergency department
EMS: emergency medical services
FAST: focused assessment with sonography for trauma
POCUS: point-of-care ultrasound
SAA: splenic artery aneurysm
